# Influence of Nutrient Medium Components on In Vitro Tuberization of *Solanum tuberosum* L. and Subsequent Minituber Production in Aeroponic and Greenhouse Conditions

**DOI:** 10.3390/life15020241

**Published:** 2025-02-05

**Authors:** Gayane Hrant Melyan, Yuri Tsatur Martirosyan, Aghvan Jumshud Sahakyan, Hovik Yakshibek Sayadyan, Andreas Shmavon Melikyan, Andranik Hakob Barseghyan, Arayik Sajan Vardanyan, Hamlet Sargis Martirosyan, Margarita Gurgen Harutyunyan, Anzhela Liparit Mkrtchyan, Inna Lendrush Hakobjanyan, Kima Seryozha Dangyan, Khachik Harut Terteryan, Kamo Atam Khazaryan, Meruzhan Haykaram Galstyan

**Affiliations:** 1 Scientific Center of Agrobiotechnology, Branch of the Armenian National Agrarian University, 1 Isi le Mulino Str., Ejmiatsin 1101, Armenia; gmggmg65@mail.ru (G.H.M.); sahakyan48@mail.ru (A.J.S.); a_melikyan@yahoo.com (A.S.M.); anbars48@rambler.ru (A.H.B.); vardanyan.arayik51@mail.ru (A.S.V.); hamlet.martirosyan.65@mail.ru (H.S.M.); margarita_harutyunyan51@mail.ru (M.G.H.); inna_hakob@yahoo.com (I.L.H.); kima.dangyan@mail.ru (K.S.D.); xachikterteryan2@gmail.com (K.H.T.); khazaryan.kamo@mail.ru (K.A.K.); galstyan.merujan@mail.ru (M.H.G.); 2 Institute of Molecular Biology, National Academy of Sciences of the Republic of Armenia (NAS RA), Hasratyan 7, Yerevan 0014, Armenia; 3 All-Russian Research Institute of Agricultural Biotechnology, Russian Academy of Agricultural Sciences, Timiryazevskaya St, 42, 117550 Moscow, Russia; yumart@yandex.ru; 4 Institute of Biochemical Physics (IBCP), RAS—Russian Academy of Sciences, Kosyagina Str. 4, 119334 Moscow, Russia; 5 Faculty of Geography and Geology, Yerevan State University, 1 Alex Manoogian, Yerevan 0025, Armenia; 6 SPS “Arm Biotechnologies” NAS RA, Yerevan 0056, Armenia; 7 Faculty of Agronomy, Armenian National Agrarian University (ANAU), Teryan 74 Str., Yerevan 0009, Armenia; a.l.m.2012@mail.ru

**Keywords:** aeroponics, in vitro tuberization, minitubers, plant growth regulators, potato

## Abstract

Potatoes, a vital global food crop, have shown remarkable adaptability, significantly contributing to food security. Technological advancements now enable their cultivation from soil-based systems to liquid synthetic nutrient media, even in artificial closed environments without natural light or fertile soil. This study examined the effects of Benzylaminopurine (BAP) and Kinetin (Kin) at concentrations ranging from 0 to 5 mg/L and sucrose concentrations ranging from 20 to 120 g/L on in vitro tuberization, focusing on microtuber size, weight, and tuberization rate. Nodal segments from virus-free ‘*Red Scarlet*’ in vitro potato plantlets were used as explants. These explants were cultured on Murashige and Skoog (MS) medium solidified with 0.5% agar. The study also compared minituber production efficiency under soil-based greenhouse and aeroponic conditions. The highest in vitro potato tuberization rate (90%) was achieved with 80 g/L sucrose and 3.0 mg/L BAP. After induction, virus-free microtubers were transferred to both greenhouse conditions and aeroponic systems for further assessment of minituber production and biochemical composition. These findings demonstrate the potential of aeroponics as a superior method for producing high-quality, pathogen-free minitubers. Aeroponics resulted in significantly higher minituber yields compared to soil-based greenhouse systems, offering a scalable and efficient solution for seed production.

## 1. Introduction

Potato minituber production is a critical method for seed potato propagation, providing high-quality, virus-free planting material [1,2,3] free from potato spindle tuber viroid (PSTVd) [4,5]. Traditionally, soil-based greenhouse systems have been the standard for this purpose. However, recent advancements in aeroponics—a soilless cultivation technique where plants are suspended in air and nourished by nutrient mist—offer promising alternatives to enhance tuberization efficiency, yield, and quality [6,7,8,9,10].

Potatoes (*Solanum tuberosum* L.), a key global food crop, thrive in diverse environments, from the highlands of Peru to arid desert regions. Advances in agricultural technologies, such as synthetic nutrient media and fully enclosed, soil- and light-free environments, expand the potential for the cultivation of potatoes in unconventional systems [11,12,13,14]. These developments not only revolutionize traditional farming but also open possibilities for growing crops in extraterrestrial environments, such as on the Moon and Mars [15].

A significant innovation in potato propagation is the production of in vitro microtubers—small tubers, often only a few hundred microns in size, that can grow into full-sized tubers while retaining the genetic traits of the parent variety [16,17]. This technique offers several advantages, including year-round cultivation and the ability to transport pathogen-free, lightweight seed material [18,19]. Furthermore, microtubers can be used to create and maintain a genetic collection of commercial potato varieties and species for breeding programs and molecular–genetic studies. However, optimizing large-scale production remains a challenge, with the sucrose concentration, cytokinin type, and photoperiod being critical factors influencing tuberization efficiency [20,21,22].

Additionally, metabolic processes such as sugar metabolism, hormonal balance, and nutrient uptake play crucial roles in tuber formation [23,24,25]. Tuberization is regulated by hormonal pathways and key genes, including *StSP6A*, *StCDF1*, and *StBEL5*, that influence sugar metabolism and hormonal balance [26,27,28].

In recent years, aeroponic systems have shown great potential in accelerating seed potato production by offering precise control over environmental conditions and nutrient delivery [29,30,31,32,33]. This approach facilitates the rapid multiplication of high-quality, pathogen-free seed potatoes while optimizing tuber yield and quality. Comparing aeroponic systems with traditional soil-based greenhouse systems is essential to understand their relative efficiencies and potential benefits.

For this study, the ‘*Red Scarlet*’ potato variety was selected due to its high market demand, superior culinary qualities, and adaptability to various growing conditions [34]. Its responsiveness to tissue culture techniques makes it an ideal candidate for evaluating innovative propagation methods. Plant growth regulators (PGRs) BAP and Kin, along with varying sucrose concentrations, were chosen based on their proven efficacy in promoting tuberization and enhancing microtuber size and weight in previous studies [35,36,37]. Both BAP and Kin are known cytokinins that promote cell division and differentiation, which are essential for successful microtuber formation [38]. Sucrose, as the primary carbon source, provides the necessary energy for tuber development [39].

This study aimed to assess the effects of BAP, Kin, and varying sucrose concentrations on in vitro tuberization of ‘*Red Scarlet*’ potatoes, comparing minituber production in soil-based greenhouse and aeroponic systems. Additionally, the research analyzed the biochemical composition of aeroponically grown minitubers, focusing on yield, uniformity, vitamin C content, sugar levels, and starch content. The findings are intended to optimize strategies for improving seed potato production efficiency, yield, and quality.

## 2. Materials and Methods

### 2.1. Research Location

This study was conducted at the Scientific Center of Agrobiotechnology, Armenian National Agrarian University, during the period of 2021–2023. Virus-free potato plantlets of the ‘*Red Scarlet*’ variety, bred by HZPC, a Dutch potato breeding company, were provided by the All-Russian Research Institute of Agricultural Biotechnology, Moscow.

The study also used aeroponic systems developed and patented at the same institute [12]. The plantlets were tested for the presence of potato viruses and viroids using diagnostic kits from Sintol (Cat. No. PV-013, Moscow, Russia) and AgroDiagnostica (Moscow, Russia), both of which specialize in plant disease diagnostics.

### 2.2. Plant Material and Culture Conditions

For this study, the ‘*Red Scarlet*’ potato variety was selected due to its high demand, excellent culinary qualities, and adaptability. Nodal segments from virus-free ‘*Red Scarlet*’ in vitro plantlets were used as explants. These explants were cultured on MS medium, solidified with 0.5% agar, and supplemented with varying concentrations of sucrose (20, 40, 60, 80, 100, and 120 g/L), as well as cytokinins—BAP and Kin—ranging from 0.0 to 5.0 mg/L, both individually and in combination. Medium without PGRs was the control.

The selection of sucrose concentrations was based on previous research indicating that carbon availability significantly influences tuberization and microtuber development in potatoes. These concentrations were chosen to optimize tuberization efficiency and microtuber size in ‘*Red Scarlet*’, a cultivar commonly used in both research and commercial potato production.

The hormonal treatments were selected based on their well-established role in promoting cell division and differentiation, crucial processes for microtuber formation. BAP and Kin have been widely used in potato tissue culture to enhance microtuber yield and size and were tested at varying concentrations to assess their impact on tuberization under controlled conditions. The medium’s pH was adjusted to 5.8 before autoclaving at 121 °C for 20 min.

### 2.3. Microtuber Induction

Microtuber induction commenced after the in vitro multiplication phase. Cultures were incubated under controlled conditions at 22 ± 1 °C with a photoperiod of 8 h light/16 h dark for 10 days. Subsequently, the plantlets were transferred to diffused light conditions for two weeks to promote tuberization. To further stimulate microtuber formation, the cultures were placed at 10 °C. Each treatment included 20 explants, cultured individually in glass test tubes, and the experiment was conducted in triplicate.

### 2.4. Assessment of Microtuber Production

Microtuber production was assessed after 70 days under different sucrose concentrations and cytokinin treatments (BAP and Kin). Key measured parameters included the number of microtubers per plantlet, microtuber diameter (mm), and microtuber weight (mg). The percentage of tuberization was calculated using the following equation:

Tuberization (%) = (Number of explants with tubers/Total number of explants) × 100

The obtained microtubers were used as planting material for both greenhouse and aeroponic systems. The aim was to assess the potential of using potato microtubers as a method for preserving genetic material and as an alternative to in vitro plantlets.

### 2.5. Use of Microtubers as Planting Material for the Aeroponic and Greenhouse Systems

Virus-free in vitro-derived ‘*Red Scarlet*’ microtubers were transferred from culture vessels to the aeroponic system. The system consisted of a closed chamber where the microtubers were secured in mesh cup holders. Under high humidity conditions, with periodic misting of the nutrient solution, the microtubers rapidly sprouted, developed roots, and grew shoots.

The nutrient solution, based on a modified Hoagland formula [40], contained essential macronutrients (N, P, K, Ca, and Mg) and micronutrients (Zn, Fe, Cu, Co, Mn, and Mo) in chelated form, along with boron in its standard form, and was delivered to the roots through high-pressure misting. Hoagland’s solution consists of the following nutrients:

- Macronutrients (in mM): nitrogen (as NO_3_^−^): 15 mM; phosphorus (as H_2_PO_4_^−^): 1 mM; potassium (as K⁺): 6 mM; calcium (as Ca^2^⁺): 5 mM; magnesium (as Mg^2^⁺): 2 mM; sulfur (as SO_4_^2−^): 2 mM.
- Micronutrients (in µM): iron (as Fe-EDTA): 100 µM; boron (as H_3_BO_3_): 50 µM; manganese (as MnSO_4_): 10 µM; zinc (as ZnSO_4_): 1 µM; copper (as CuSO_4_): 0.5 µM; molybdenum (as Na_2_MoO_4_): 0.05 µM.

The pH of the nutrient solution was maintained at 5.7 ± 0.1. The salt concentration was monitored using an electrical conductivity sensor and maintained at 1.8–2.5 mS/cm. Irrigation was controlled using a humidity sensor located inside the chamber. When the humidity level decreased to 45%, the pump was automatically activated, and a fine-mist aerosol was sprayed. The aerosol particle diameter ranged from 5 to 125 microns. Once the humidity reached 65–70%, the pump automatically turned off. These conditions created optimal growth conditions for shoot and root development and nutrient absorption. The plants were kept in the aeroponic system under controlled conditions at 22 ± 2 °C with a 16 h light/8 h dark photoperiod. Root development and tuber formation were continuously monitored. Plant growth and tuber development were assessed over 90 days, with data recorded on the size, number, and weight of the tubers.

Following in vitro microtuber induction, the tubers were transferred to both greenhouse conditions and an aeroponic system for further minituber production. In the greenhouse, the microtubers were planted in individual 3 L pots filled with a soil mix consisting of 50% peat, 30% sand, and 20% perlite. The pots were placed in a greenhouse maintained at 22 ± 2 °C, with a 16 h light/8 h dark photoperiod and 60–70% relative humidity.

The plants were regularly watered to maintain soil moisture and fertilized every two weeks with water-soluble fertilizers from Yara (Norway).

1. First Growth Stage (from mass germination to the beginning of budding)

The nutrient solution was prepared using YaraTera Kristalon (NPK 18-18-18 + micro) at 1 g/L, sourced from yara International, Oslo, NorwayDone, with the addition of 0.5 g/L YaraTera Calcinit (Ca(NO_3_)_2_) and 0.3 g/L magnesium nitrate (Mg(NO_3_)_2_).

2. Second Growth Stage (from the start of budding to the beginning of flowering)

For this stage, the nutrient solution was adjusted to YaraTera Kristalon Yellow (NPK 13-40-13 + micro) at 1 g/L, along with 0.5 g/L calcium nitrate (Ca(NO_3_)_2_) and 0.3 g/L magnesium nitrate (Mg(NO_3_)_2_).

3. Third Growth Stage (from the beginning of flowering to the start of wilting of the foliage)

In the final stage, a nutrient solution containing YaraTera Kristalon Brown (NPK 3-11-38 + micro) at 2 g/L was used, supplemented with 1 g/L calcium nitrate (Ca(NO_3_)_2_) and 0.7 g/L magnesium nitrate (Mg(NO_3_)_2_).

Both the greenhouse and aeroponic systems were evaluated for their effects on minituber development and composition. Minituber production followed a consistent schedule across the three years:

- 2021: Planting occurred on 15 April, utilizing microtubers in both greenhouse and aeroponic systems. Harvest was completed on 14 July.
- 2022: Planting commenced on 18 April, again employing microtubers in both greenhouse and aeroponic systems. Harvest concluded on 17 July.
- 2023: Planting began on 10 April, with microtubers planted in both greenhouse and aeroponic systems. Harvest was finalized on 9 July.

### 2.6. Nutrient Composition of Potato Tubers

To assess the influence of growing conditions on tuber quality, a comprehensive analysis of the nutritional profiles of potato tubers grown in aeroponic and greenhouse systems was conducted. Both macronutrient and micronutrient content in the tubers from each system were measured.

### 2.7. Biochemical Analysis of Minitubers

After harvesting from both the greenhouse and aeroponic systems, several biochemical analyses were conducted on the minitubers to evaluate their composition. The analyses included the measurement of dry matter content, starch, vitamin C, sugar levels, and nitrate content. The following methods were used:

- Dry matter content was measured using the thermogravimetric method, according to which samples were dried to a constant weight at 105 °C (ISO 1026:1982) [41].
- Sugar content was assessed using Bertrand’s method, which involves the reduction of copper (II) ions to copper (I) oxide under alkaline conditions (AOAC Official Method 923.09) [42].
- Vitamin C content was determined using UV spectrophotometry, following the titration of ascorbic acid with a dye (Negi, 2022) [43].
- Nitrate content was analyzed using the Griess reagent method, which involves the diazotization of nitrite with sulfanilamide and subsequent coupling with N-(1-naphthyl) ethylenediamine dihydrochloride to form a colored azo dye (METTLER TOLEDO, n.d.) [44].
- Starch content was evaluated using the enzymatic colorimetric method, whereby starch is enzymatically hydrolyzed to glucose and the glucose is quantified (AOAC Official Method 996.11) [45].

### 2.8. Statistical Analysis

All data are expressed as mean ± standard deviation (SD). Statistical significance was considered at a *p*-value < 0.05. Significant differences between the means were determined using Student’s *t*-test, performed using GraphPad Prism version 9.5.0.

## 3. Results

### 3.1. Effect of Sucrose Concentration on Microtuber Induction and Characteristics

**Figure 1:**
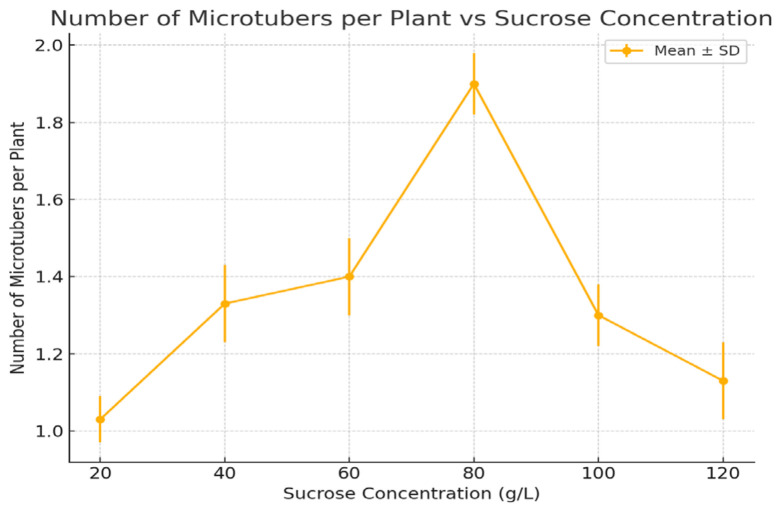
Average number of microtubers per plant at different sucrose concentrations (2021–2023 averages). Error bars represent standard deviations. Statistical analysis using a *t*-test revealed significant differences between treatments (*p* < 0.05).

**Figure 2:**
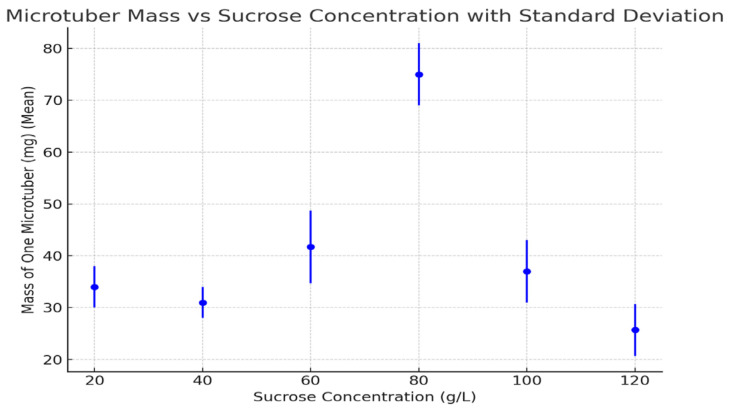
Mass of one microtubers under different sucrose concentrations (2021–2023 averages). Error bars represent standard deviations. Statistical analysis using a *t*-test revealed significant differences between treatments (*p* < 0.05).

**Figure 3:**
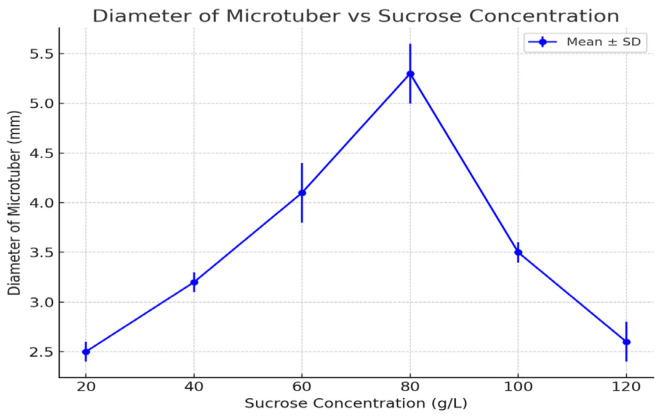
Diameter of microtubers under different sucrose concentrations (2021–2023 averages). Error bars represent standard deviations. Statistical analysis using a *t*-test revealed significant differences between treatments (*p* < 0.05).

The in vitro effect of different sucrose concentrations on microtuber induction and characteristics was examined. Table 1 presents the results, while Figure 1, Figure 2 and Figure 3 illustrate the effects of sucrose on the following key parameters: the number of microtubers per plant (Figure 1), microtuber mass (Figure 2), and microtuber diameter (Figure 3).

### 3.2. Percentage of Plants Induced to Form Microtubers

The percentage of plants that produced microtubers increased with sucrose concentration, peaking at 80 g/L, at which point 63.3% of plants formed microtubers. Beyond this peak, the percentage slightly decreased at 100 g/L (50.4%) and further declined at 120 g/L (46.6%). The trend indicates that the sucrose concentration significantly impacted microtuber induction, with 80 g/L being the optimal concentration.

Higher concentrations, particularly 100 g/L and 120 g/L, resulted in a decrease in induction efficiency, likely due to osmotic stress or the inhibitory effect of elevated sugar levels on tuberization.

### 3.3. Number of Microtubers per Plant

The number of microtubers per plant increased with sucrose concentration, with the highest number (1.9 microtubers per plant) observed at 80 g/L. A noticeable decrease in microtuber number was observed at both lower (20 g/L, 1.03 microtubers) and higher concentrations (120 g/L, 1.13 microtubers).

This suggested that intermediate sucrose concentrations (around 80 g/L) were most favorable for microtuber formation, promoting tuber initiation. In contrast, both insufficient (20 g/L) and excessive (120 g/L) sucrose concentrations appeared to inhibit microtuber formation, indicating the importance of optimal carbohydrate availability for effective tuber development.

### 3.4. Mass of One Microtuber

The mass of individual microtubers was greatest at 80 g/L (75 mg), with significantly smaller tubers observed at other concentrations, particularly at 120 g/L, at which point the mass was only 25.7 mg. Lower sucrose concentrations (20 g/L and 40 g/L) also produced relatively small microtubers.

The mass of individual microtubers showed a clear correlation with sucrose concentration, with the peak mass at 80 g/L corresponding to the highest percentage of induction and the greatest number of microtubers per plant. The reduced mass at both extreme sucrose concentrations (either too low or too high) was likely due to insufficient carbohydrate supply or osmotic stress, which hindered proper tuber growth.

### 3.5. Diameter of Microtubers

The diameter of microtubers followed a trend similar to that of mass, with the largest diameters observed at 80 g/L (5.3 mm).

Smaller diameters were observed at both lower and higher sucrose concentrations, with diameters of 2.5 mm at 20 g/L and 2.6 mm at 120 g/L. The diameter of microtubers reflected overall growth, with 80 g/L providing the optimal conditions for tuber formation and expansion. Lower sucrose concentrations likely limited tuber expansion due to an insufficient carbohydrate supply, while higher concentrations may have induced osmotic stress, restricting growth.

### 3.6. Effect of Cytokinins on Tuber Formation

After identifying 80 g/L sucrose as the optimal concentration for microtuberization, cytokinins—specifically BAP and Kin—were tested to evaluate their impact on tuber formation. As shown in Table 2, cytokinin treatments enhanced tuberization rates compared to the control (55.5%), with the highest tuberization rates observed at 3 mg/L BAP (90%) and 4 mg/L Kin (80.5%).

For BAP treatments, tuberization increased steadily with concentration, peaking at 3.00 mg/L, at which point the highest tuberization rate of 90.0% was observed. However, concentrations above 3.00 mg/L (4.00 and 5.00 mg/L) led to a significant decline in tuberization rates (dropping to 55.0% and 36.5%, respectively). This suggests that higher concentrations of BAP beyond the optimal level inhibit tuber formation.

Similarly, Kin treatments resulted in an increase in tuberization percentage as the concentration rose, with the maximum rate of 80.5% at 4.00 mg/L. At the highest concentration (5.00 mg/L), the tuberization rate dropped to 46.6%, mirroring the inhibitory effect observed with higher BAP concentrations.

Both cytokinins demonstrated clear optimal concentrations for tuberization, with 3.00 mg/L BAP and 4.00 mg/L Kin yielding the highest tuberization rates. Beyond these concentrations, tuberization decreased, indicating that excessive levels of either cytokinin may have an inhibitory effect on tuber formation.

The effects of these phytohormonal treatments on microtuber growth characteristics are further illustrated in Table 3, providing a comprehensive overview of the data.

- BAP: A steady increase in the number of microtubers per shoot was observed as BAP concentrations increased. Starting with the control (1.60 microtubers per shoot), the number nearly tripled at the peak concentration of 3.0 mg/L, reaching 4.60 microtubers per shoot. After this peak, a decline in performance was noted at higher concentrations.

Regarding microtuber weight, BAP significantly increased the weight of microtubers, with the control producing 60.0 ± 10.0 mg and 3.0 mg/L BAP yielding a remarkable 410.0 ± 40.0 mg. This represents nearly a seven-fold increase compared to the control. However, concentrations higher than 3.0 mg/L resulted in a decline in weight.

The microtuber diameter followed a similar upward trend, with the largest diameter (8.43 ± 0.20 mm) recorded at 3.0 mg/L BAP, compared to the control’s 5.30 ± 0.10 mm. Concentrations above 3.0 mg/L led to a reduction in tuber size.

- Kin: While Kin also promoted microtuber formation, the increase was more modest compared to BAP. At 3.0 mg/L Kin, the number of microtubers peaked at 4.00 ± 0.10 per shoot. As concentrations increased further, a noticeable decline in the number of microtubers was observed. The microtuber weight in Kin treatments did not reach the same levels as those observed with BAP. The highest weight (140.0 ± 10.0 mg) was observed at 4.0 mg/L Kin. However, as with BAP, higher concentrations led to a decrease in weight.

In terms of microtuber diameter, Kin had a weaker effect, with the largest diameter observed at 3.0 mg/L (4.50 ± 0.20 mm), which was smaller compared to the results observed with BAP.

A positive correlation was observed between the number of microtubers per shoot, their weight, and their diameter for both phytohormones (BAP and Kin). As the number of microtubers increased, their weight and diameter also increased, particularly under BAP treatment. This suggests that higher concentrations of BAP and Kin generally promoted enhanced growth. However, excessive concentrations—especially those above 3.0 mg/L for BAP and 5.0 mg/L for Kin—resulted in a decline in both the number and size of microtubers, indicating diminishing returns.

**Figure 4:**
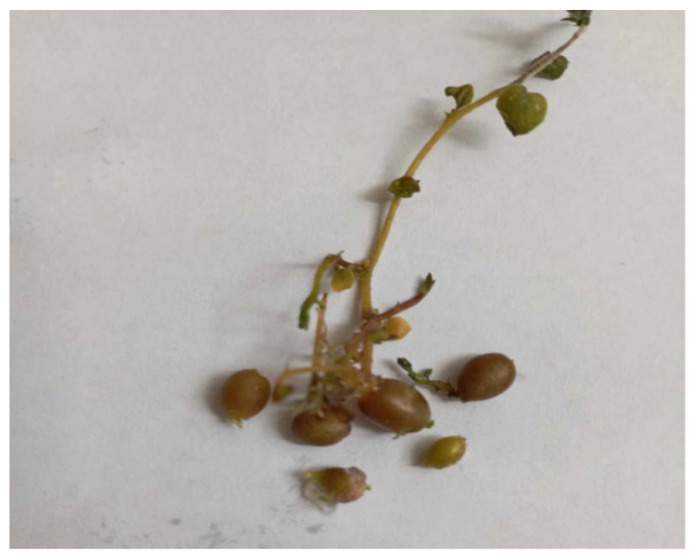
In vitro microtuber development of the ‘*Red Scarlet*’ potato variety under treatment with BAP (3.0 mg/L) and sucrose (80 g/L).

Cytokinins stimulated the microtuberization process by transforming upright shoots into horizontal stolons, which then grew downward to form tubers (Figure 4).

A 3.0 mg/L BAP concentrated produced the highest values for the number of microtubers per shoot, fresh weight, and diameter (4.60 microtubers per shoot, 410.0 ± 40.0 mg, and 8.43 ± 0.20 mm, respectively), followed by the 2.5 mg/L BAP treatment (4.10 microtubers per shoot, 350.0 ± 20.0 mg, and 7.45 ± 0.05 mm, respectively).

### 3.7. Evaluation of Potato Growth Metrics and Survival Rates in Greenhouse and Aeroponic Systems

The survival rate and key potato growth metrics were evaluated across both greenhouse and aeroponic systems to assess their efficiency and suitability for large-scale potato production (Table 4).

As shown in Table 4, the survival rate of minitubers was higher in the aeroponic system (95%) compared to the greenhouse system (80%). Despite the slightly lower survival rate in the greenhouse, these results demonstrate strong adaptability, indicating that greenhouse conditions are also effective for healthy minituber production when using microtubers as starting material. The slight decrease in survival in the greenhouse may be attributed to differences in root zone aeration and humidity control compared to the aeroponic system.

The number of stems per microtuber was greater in the greenhouse system (3.2) than in the aeroponic system (2.1), indicating that soil-based conditions support more robust stem growth. However, the aeroponic system produced a significantly higher number of minitubers per microtuber (32.4 ± 2.6) compared to the greenhouse system (7.5 ± 1.9). Additionally, the percentage of tubers smaller than 25 mm was higher in the aeroponic system (82 ± 4.0%) compared to the greenhouse system (26 ± 5.4%). Although fewer stems were formed under aeroponic conditions compared to greenhouse conditions, this lower stem number facilitated the formation and functioning of a significantly larger number of stolons, resulting in more productive tuber formation.

In aeroponic systems, the potato plant canopy can be controlled during cultivation by redirecting shoots toward either stem formation or stolon development in the root zone.

Throughout the vegetative period, it is possible to adjust the ratio between the aboveground and underground portions of the potato plant by regulating factors such as light (intensity and spectrum), temperature (day/night gradient), and mineral nutrition (composition and ratio of chemical elements). By managing these factors, metabolites can be directed from the leaves to the root zone, particularly toward the stolons, thereby controlling the rate and quantity of minituber formation. One significant advantage of an aeroponic system is the ability to periodically harvest conditioned minitubers of a predetermined size.

This comparison highlights the advantages of an aeroponic system in promoting tuberization and producing a greater number of smaller tubers, while a greenhouse system supports better stem growth. These findings suggest that aeroponic systems may be more efficient for the production of high-quality, pathogen-free minitubers, whereas greenhouse systems may be better suited for producing larger tubers.

The evaluation of tuber size distribution in both systems revealed significant differences in the proportions of smaller and larger tubers. In the aeroponic system, a high percentage of tubers was smaller than 25 mm (82 ± 4.0%), indicating that an aeroponic environment favors the development of smaller, faster-growing tubers, likely due to improved root oxygenation and efficient nutrient absorption. However, the percentage of larger tubers (>25 mm) was considerably lower, at 18 ± 5.2%, suggesting that while an aeroponic system promotes a high rate of minituber production, it may not be as favorable for the development of larger tubers. This may be due to the controlled conditions encouraging rapid and uniform growth.

In contrast, the greenhouse system produced fewer small tubers (26 ± 5.4%) and a significantly higher percentage of larger tubers (>25 mm) at 74 ± 8.3%. This suggests that the stable, natural conditions in a greenhouse are more conducive to the development of larger minitubers, which is advantageous for large-scale potato production.

**Figure 5:**
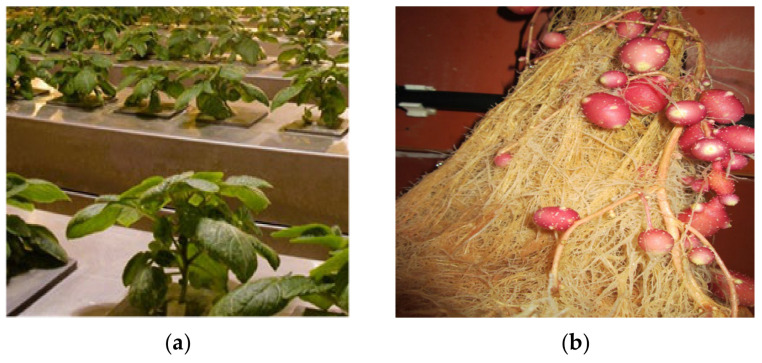
Growth of ‘*Red Scarlet*’ potato plants (**a**) and minitubers (**b**) in an aeroponic system.

**Figure 6:**
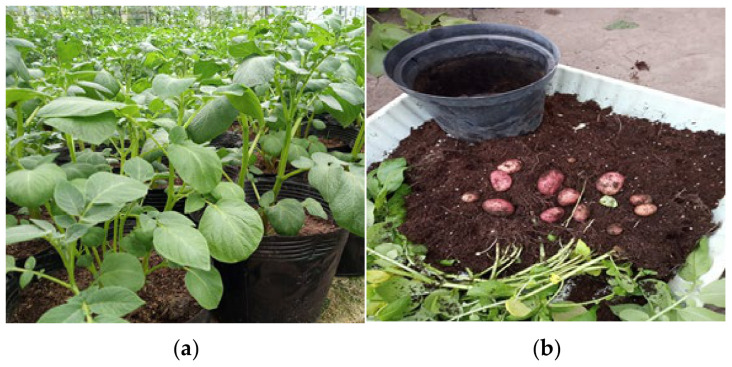
Growth of ‘*Red Scarlet*’ potato plants (**a**) and minitubers (**b**) in a greenhouse system.

Plants and minitubers produced in controlled aeroponic and greenhouse systems are illustrated in Figure 5a,b and Figure 6a,b, respectively.

### 3.8. Comparative Analysis of Potato Minituber Composition in Aeroponic and Greenhouse Systems

A comparative analysis of potato minitubers grown in aeroponic and greenhouse conditions was conducted to evaluate the impact of cultivation systems on their biochemical characteristics. The analysis revealed distinct differences in dry matter content, reducing sugars, vitamin C levels, nitrate levels, starch content, and amylose content between the two systems. A thorough analysis of the biochemical composition of potato minitubers cultivated in both aeroponic and greenhouse systems is illustrated in Table 5.

Tubers produced in the aeroponic system exhibited a lower dry matter content (16.9 ± 1.5%) compared to those grown in the greenhouse (19.8 ± 1.2%). This significant difference (*p* < 0.05) suggests that an aeroponic environment may promote better moisture retention, making it less conducive to water loss.

In terms of sugar content, the aeroponically grown tubers had a notably higher reducing sugars content (1.02 ± 0.03%) than those grown in the greenhouse (0.36 ± 0.02%), with the difference being statistically significant (*p* < 0.05). This indicates that an aeroponic system may enhance the accumulation of sugars, possibly due to more efficient nutrient delivery.

Vitamin C content was significantly higher in tubers from the aeroponic system (18.5 ± 1.0 mg/100 g) compared to those grown in the greenhouse system (16.3 ± 0.8 mg/100 g), with a statistically significant difference (*p* < 0.05). This suggests that aeroponic conditions might enhance the synthesis of ascorbic acid.

Nitrate content was lower in tubers from the aeroponic system (45.3 ± 3.5 mg/kg) compared to those from the greenhouse system (49.7 ± 2.8 mg/kg), with a statistically significant difference (*p* < 0.05). This implies that an aeroponic system may facilitate more efficient nitrate utilization or reduce nitrate accumulation.

Starch content was significantly higher in tubers grown in the greenhouse system (17.8 ± 1.0%) compared to those from the aeroponic system (10.3 ± 0.9%), with a statistically significant difference (*p* < 0.05). This suggests that greenhouse conditions may promote starch accumulation, which could be advantageous for certain cultivation purposes. In contrast, amylose content did not differ significantly between the two systems, with the aeroponic system showing 27.9 ± 1.5% and the greenhouse system showing 28.1 ± 1.1%, indicating no statistically significant difference (*p* > 0.05). This suggests that the cultivation system does not have a significant effect on amylose content in minitubers.

Overall, these findings underscore the distinct biochemical profiles of potato minitubers cultivated under different systems. An aeroponic system enhances certain nutritional factors, such as sugar and vitamin C content, while a greenhouse system favors higher starch accumulation. Understanding these differences provides valuable insights for the selection of the most suitable cultivation method based on specific production goals and nutritional requirements.

## 4. Discussion

This study extensively examined the impact of varying sucrose concentrations, growth regulators, and cultivation techniques on potato microtuber initiation and development. Our findings highlight the critical role of sucrose in optimizing microtuber production, with higher concentrations significantly enhancing induction rates. However, they also demonstrate the complexity of achieving an optimal balance between carbohydrate availability and osmotic pressure.

At 80 g/L sucrose, the highest induction rate of 63.3% was observed, with an average tuber size of 5.3 mm in diameter and a mass of 0.065 g. This result is consistent with the findings of Wazir et al. (2015) and Fufa and Diro (2014) [46,47], who reported similar sucrose concentrations for optimal microtuber formation. The physiological basis for this observation likely lies in the balance between providing sufficient carbohydrates for rapid cell expansion and avoiding osmotic stress, as suggested by Cui et al. (2010) [48]. Osmotic stress is known to hinder crucial physiological processes, such as cell elongation and division, as noted by Askari (2023) [49].

Interestingly, our findings diverged slightly from those of Mohamed and Girgis (2023) [50], who observed optimal microtuber induction at a higher sucrose concentration (90 g/L). This suggests that the sucrose threshold for microtuberization might be cultivar-specific or influenced by environmental factors such as temperature and light conditions.

In our study, higher sucrose concentrations (100–120 g/L) resulted in reduced induction rates, supporting the idea that excessive sucrose levels create osmotic stress, hindering cell division and expansion and leading to smaller tubers. This pattern aligns with previous reports indicating that excessive sugar levels may inhibit tuber organogenesis [51,52].

Moderate sucrose levels (40–60 g/L) produced balanced outcomes, yielding higher induction rates with moderately sized tubers. These results are consistent with those of Hossain et al. (2017) [53], who found that moderate sucrose concentrations optimize energy availability for tuber formation. At the lower concentration of 20 g/L, the significant drop in induction rates (10%) and smaller tuber size reaffirm the importance of carbohydrate availability during tuberization [54,55].

These observations suggest that although lower sucrose concentrations reduce competition for carbohydrates, they still limit overall growth potential, as noted in similar studies [50]. Additionally, the higher tuber mass at lower sucrose concentrations may be due to reduced competition for the limited available carbohydrates, resulting in fewer but larger tubers [55]. The peak performance at 80 g/L indicates that this concentration provides an ideal balance between carbohydrate availability and osmotic pressure [55], supporting cell division and expansion, both of which are critical for tuber growth [56]. Conversely, sucrose concentrations above 80 g/L reduced induction rates and tuber size, likely due to osmotic stress and reduced cellular turgor [52].

In terms of growth regulators, BAP (3.0 mg/L) proved to be the most effective in enhancing all microtuber growth parameters, including tuber number, weight, and diameter. This finding confirms the observations of Sosnowski et al. (2023) [57] and Mohamed et al. (2023) [50], who reported optimal tuber production with cytokinin concentrations. Our results, however, indicate that a slightly lower concentration of BAP (3.0 mg/L) is sufficient to achieve significant improvements in tuberization, contrasting with studies suggesting higher cytokinin levels (4.0–5.0 mg/L) [57]. This discrepancy might be attributed to differences in potato cultivars or variations in environmental conditions during the experiments. While kinetin (Kin) also stimulated microtuber formation, it was less effective than BAP, particularly regarding tuber weight and diameter, supporting the view that BAP is a more potent growth regulator in terms of promoting tuberization.

The comparison between aeroponic and greenhouse systems revealed distinct advantages. The aeroponic system resulted in a significantly higher number of smaller tubers (32.4 minitubers per plant) compared to the greenhouse system (7.5 per plant). This difference is likely attributed to improved aeration and nutrient availability in aeroponics, accelerating tuberization [58,59,60]. However, the tubers grown in the greenhouse system were larger, with over 74% of them exceeding 25 mm. This suggests that soil-based systems may be more conducive to the development of larger tubers with greater starch reserves [61,62].

Our research aimed to optimize key environmental factors influencing plant growth and development. These factors behave differently in aeroponic and greenhouse systems, where root growth, stolon formation, and tuberization occur in soil in the greenhouse but in an oxygen-rich air environment without soil in aeroponics. Therefore, the results of this study, specifically with respect to the quantity and quality of minitubers, must be analyzed within the context of planting time, the duration of the growing period, and the stage of tuber development at harvest.

Biochemical analysis further revealed that aeroponically grown minitubers accumulated lower amounts of dry matter, starch, and crude protein but higher levels of reducing sugars and vitamin C compared to greenhouse-grown minitubers. These differences are likely due to the shorter growing period in aeroponic systems [58,59,60], which limits starch accumulation but enhances metabolic activity and antioxidant defenses. In contrast, greenhouse-grown tubers benefited from a longer growing period, allowing for greater starch and protein accumulation, which is essential for storage and delayed sprouting. Additionally, the extended growing period in the greenhouse facilitated higher nitrate nitrogen content, which could be attributed to more extensive nutrient absorption [61,62].

Aeroponic minituber production relies on the regulation of donor–acceptor relationships between the leaves (donors) and developing tubers (acceptors). Timely removal of tubers of specific sizes promotes nutrient flow from the leaves to the stolons, stimulating further tuberization [63].

Despite the advantages, aeroponics faces challenges such as high initial setup costs, technical expertise requirements, and energy demands, which could limit its adoption, particularly for small-scale farmers [64,65]. Additionally, the scalability of aeroponics is a concern due to the need for highly controlled environments, limiting its use in regions lacking necessary infrastructure [30].

### 4.1. Incorporating Recent Studies on Controlled Environment Agriculture (CEA)

Recent research in controlled environment agriculture (CEA) emphasizes the potential of aeroponic systems to improve both crop yields and tuber quality. Studies have shown that CEA systems, including aeroponic cultivation systems, offer promising benefits in terms of producing high-density minitubers with faster growth rates compared to traditional soil-based systems [66,67], primarily due to optimization of environmental controls, such as light intensity, temperature, and nutrient composition [65,68].

### 4.2. Economic Efficiency and Environmental Sustainability

While aeroponics offers precise environmental control and year-round production of high-quality, pathogen-free minitubers, it faces economic challenges, such as high equipment costs and energy demands [69]. However, its benefits are notable in regions with limited water resources or poor soil quality, where aeroponics can significantly improve water efficiency and nutrient uptake [70,71]. These environmental benefits could make aeroponics an attractive option for regions facing climate change challenges or agricultural resource constraints.

### 4.3. Directions for Future Research

Future studies should focus on the field performance of aeroponically grown minitubers, examining yield, storage capacity, and environmental stress resistance. Additionally, research into genetic factors influencing minituber formation in various potato varieties can optimize cultivation practices and improve minituber production across different genetic backgrounds.

### 4.4. Practical Recommendations

- For producers, we recommend the use of sucrose concentrations of approximately 80 g/L and 3.0 mg/L BAP to optimize minituber production. Aeroponic systems are ideal for high-density minituber production, while greenhouse systems are better suited for producing larger tubers intended for field planting.
- We recommend that researchers focus on optimizing sucrose concentrations and hormonal treatments for different potato varieties and investigate the effects of additional phytohormones, such as gibberellins and other less studied hormones, to enhance in vitro microtuber formation, followed by the propagation of these microtubers to improve minituber quality in greenhouse and aeroponic systems
- We recommend that policymakers invest in aeroponic and greenhouse technologies, as well as training and energy-saving systems, to improve minituber production. Standardized guidelines for sucrose and growth regulator use would simplify seed potato production protocols.

## 5. Conclusions

This study demonstrated that pathogen-free microtubers produced under in vitro conditions can be successfully propagated into high-quality minitubers using both aeroponic and traditional greenhouse cultivation methods. While some differences in the biochemical composition of minitubers were observed between those grown aeroponically and those grown in soil-based greenhouse systems, aeroponics offers a distinct advantage in terms of producing more uniform minitubers in higher quantities. This makes aeroponics particularly valuable for large-scale seed potato production. The uniformity and higher output contribute to the efficiency and scalability of seed production, accelerating the adoption of new potato varieties in agriculture.

Further research is needed to conduct a comparative evaluation of minitubers produced using aeroponics and those grown in traditional greenhouse systems, with a focus on yield potential, storage capacity, environmental stress tolerance, and resistance to secondary infections by potato pathogens. Additionally, optimizing or developing new agronomic technologies specifically for seed potato production based on both aeroponically and greenhouse-grown minitubers is essential to fully harness the advantages of both systems.

## Figures and Tables

**Table 1:** Effect of sucrose concentration on microtuber induction in ‘*Red Scarlet*’ potato (*Solanum tuberosum* L.): percentage of plants induced and number, mass, and diameter of microtubers (2021–2023 averages).

Sucrose Concentration (g/L)	Percentage of Plants Induced to form Microtubers (%)	Number of Microtubers per Plant (Mean ± SD)	Mass of One Microtuber (mg) (Mean ± SD)	Diameter of One Microtuber (mm) (Mean ± SD)
20	10.0 ± 2.1 ^d^	1.03 ± 0.06 ^c^	34.0 ± 4.0 ^b^	2.5 ± 0.1 ^e^
40	38.0 ± 3.5 ^c^	1.33 ± 0.1 ^b^	31.0 ± 3.0 ^b^	3.2 ± 0.1 ^d^
60	46.5 ± 3.0 ^b^	1.40 ± 0.1 ^b^	41.7 ± 7.0 ^b^	4.1 ± 0.3 ^b^
80	63.3 ± 4.2 ^a^	1.90 ± 0.08 ^a^	75.0 ± 6.0 ^a^	5.3 ± 0.3 ^a^
100	50.4 ± 2.8 ^b^	1.30 ± 0.08 ^b^	37.0 ± 6.0 ^b^	3.5 ± 0.1 ^c^
120	46.6 ± 3.1 ^b^	1.13 ± 0.1 ^c^	25.7 ± 5.0 ^c^	2.6 ± 0.2 ^e^

Note: Different letters within the same column indicate statistically significant differences (*p* < 0.05) between groups based on Student’s *t*-test. Values are presented with a 95% confidence interval.

**Table 2:** Effects of BAP and Kin on the tuberization rate of the ‘*Red Scarlet*’ potato variety under 80 g/L sucrose (2021–2023 averages).

Treatment	Cytokinin Concentration (mg/L)	In Vitro Tuberization (%)
BAP	0 (control)	55.5 ^e^
	0.25	56.7 ^e^
	0.5	60.0 ^de^
	1.0	66.6 ^de^
	2.0	70.0 ^c^
	2.5	75.0 ^c^
	3.0	90.0 ^a^
	4.0	55.0 ^e^
	5.0	36.5 ^f^
Kin	0.25	56.6 ^e^
	0.5	60.0 ^e^
	1.0	66.7 ^d^
	2.0	70.0 ^cd^
	2.5	71.7 ^c^
	3.0	76.5 ^c^
	4.0	80.5 ^b^
	5.0	46.6 ^f^

Note: Values with different letters are significantly different (*p* < 0.05) based on student’s *t*-test. All values are reported with a 95% confidence interval.

**Table 3:** Analysis of the effects of phytohormonal treatments on microtuber growth characteristics (2021–2023 averages).

Treatment	Phytohormone Concentration (mg/L)	Microtubers per Shoot (Mean ± SD)	Weight of One Microtuber (mg, Mean ± SD)	Diameter of one Microtuber (mm, Mean ± SD)
Control	0.00	1.60 ± 0.10 ^h^	60.0 ± 10.0 ^ef^	5.30 ± 0.10 ^e^
BAP	0.25	1.95 ± 0.08 ^g^	90.0 ± 10.0 ^e^	6.25 ± 0.05 ^d^
	0.50	2.30 ± 0.08 ^f^	130.0 ± 10.0 ^d^	6.67 ± 0.15 ^c^
	1.0	2.95 ± 0.10 ^e^	150.0 ± 20.0 ^d^	6.80 ± 0.10 ^c^
	2.0	3.48 ± 0.15 ^d^	260.0 ± 20.0 ^c^	7.17 ± 0.15 ^b^
	2.5	4.10 ± 0.15 ^b^	350.0 ± 20.0 ^b^	7.45 ± 0.05 ^b^
	3.0	4.60 ± 0.10 ^a^	410.0 ± 20.0 ^a^	8.43 ± 0.20 ^a^
	4.0	3.70 ± 0.14 ^c^	370.0 ± 10.0 ^b^	6.50 ± 0.38 ^c^
	5.0	1.40 ± 0.15 ^h^	50.0 ± 20.0 ^f^	4.50 ± 0.20 ^f^
Kin	0.25	2.50 ± 0.14 ^f^	70.0 ± 10.0 ^ef^	4.15 ± 0.10 ^f^
	0.50	2.75 ± 0.16 ^e^	80.0 ± 20.0 ^ef^	4.25 ± 0.12 ^f^
	1.0	3.40 ± 0.15 ^d^	90.0 ± 10.0 ^e^	4.43 ± 0.15 ^f^
	2.0	3.85 ± 0.10 ^c^	100.0 ± 20.0 ^d^	4.10 ± 0.26 ^f^
	2.5	3.65 ± 0.16 ^cd^	120.0 ± 20.0 ^d^	4.20 ± 0.26 ^f^
	3.0	4.00 ± 0.10 ^b^	110.0 ± 20.0 ^d^	4.50 ± 0.20 ^f^
	4.0	3.40 ± 0.08 ^d^	140.0 ± 10.0 ^d^	4.00 ± 0.10 ^g^
	5.0	2.10 ± 0.16 ^g^	90.0 ± 10.0 ^e^	3.13 ± 0.20 ^h^

Note: Different letters within each column indicate statistically significant differences (*p* < 0.05) between treatment groups based on student’s *t*-test, with a 95% confidence level.

**Table 4:** Performance of minitubers derived from in vitro microtubers of the ‘*Red Scarlet*’ potato variety in greenhouse and aeroponic systems (2021–2023 averages).

Potato Variety	Origin of Mini Tubers	Survival Rate (%) (Mean ± SD)	Number of Stems per Microtuber (Mean ± SD)	Number of Mini Tubers per Microtuber (Mean ± SD)	Number of Tubers (%) <25 mm (Mean ± SD)
‘*Red Scarlet*’	Aeroponic	95.0 ± 4.08 ^a^	2.1 ± 0.14 ^b^	32.4 ± 2.6 ^a^	82 ± 4.0 ^a^
	Greenhouse	80.0 ± 4.08 ^b^	3.2 ± 0.18 ^a^	7.5 ± 1.9 ^b^	26 ± 5.4 ^b^

Note: Means followed by different letters within each column are significantly different (*p* < 0.05, 95% confidence) based on student’s *t*-test with standard deviation.

**Table 5:** Biochemical composition of potato minitubers grown in aeroponic and greenhouse systems (2021–2023 averages).

Biochemical Parameter	Aeroponic System (Mean ± SD)	Greenhouse System (Mean ± SD)	Statistical Significance
Dry Matter Content (%)	16.9 ± 1.5	19.8 ± 1.2	*p* < 0.05
Reducing Sugars Content (%)	1.02 ± 0.03	0.36 ± 0.02	*p* < 0.05
Vitamin C Content (mg/100 g)	18.5 ± 1.0	16.3 ± 0.8	*p* < 0.05
Nitrate Content (mg/kg)	45.3 ± 3.5	49.7 ± 2.8	*p* < 0.05
Starch Content (%)	10.3 ± 0.9	17.8 ± 1.0	*p* < 0.05
Amylose Content (%)	27.9 ± 1.5	28.1 ± 1.1	*p* > 0.05
Crude Protein Content (%)	1.93 ± 0.24	2.43 ± 0.19	*p* < 0.05
Total Protein Content (%)	0.98 ± 0.03	1.05 ± 0.04	*p* < 0.05

Note: Statistical significance between the aeroponic and greenhouse systems was evaluated using student’s *t*-test at a 95% confidence level (*p* < 0.05).

## Data Availability

The data that support the findings of this study are available from the corresponding author upon reasonable request.
